# Supplementary material for: Broadband Negative Refraction of Highly Squeezed Hyperbolic Polaritons in 2D Materials
Source: Research (Wash D C). 2018 Dec 19;2018:2532819. doi: 10.1155/2018/2532819 (PMC6750088; doi:10.1155/2018/2532819)
Supplement: Supplementary Materials — Section S1: Dispersion of hybrid polaritons supported by anisotropic metasurfaces. Section S2: All-angle negative refraction of hyperbolic graphene plasmons. Section S3: Loss influence on the bandwidth having Im(σxx)∙Im(σyy) < 0. Fig. S1. Real part of effective surface conductivity of graphene metasurface. Fig. S2. Full width at half maximum of the image for the point source in Figure 1(c). Fig. S3. All-angle negative refraction of hyperbolic polaritons when the real material loss is considered. Fig. S4. Isofrequency contours of hyperbolic graphene plasmons at 10 THz, 15 THz and 20 THz. Fig. S5. Isofrequency contours of hyperbolic graphene plasmons at 1 THz and 40 THz. Fig. S6. Substrate influence on the all-angle negative refraction of hyperbolic graphene plasmons at 15 THz. Fig. S7. All-angle negative refraction of hyperbolic polaritons in nanostructures of patterned 2D materials with a pitch of L = 100 nm. Fig. S8. Loss influence on the bandwidth having Im(σxx,l)∙Im(σyy,l) < 0. [file 2532819.f1.docx]

**Supplementary materials for**

**“Broadband negative refraction of highly squeezed hyperbolic polaritons in 2D materials”**

**Section S1: Dispersion of hybrid polaritons supported by anisotropic metasurfaces**

Contrary to the isotropic metasurface (i.e., $\sigma=[\sigma_{xx},\sigma_{yy}]$ and $\sigma_{xx}=\sigma_{yy}$) which supports the propagation of either transverse-magnetic (TM) (*39, 40*) or transverse-electric (TE) polaritons (*41-43*), the anisotropic metasurface (i.e., $\sigma_{xx}\neq\sigma_{yy}$) supports the hybrid TM-TE polaritons (*20, 24, 45-47*). Below we analytically solve the dispersion of these hybrid polaritons. We assume the anisotropic metasurface located at the interface (i.e., the plane of $z=0$) between region 1 ($z>0$, air) and region 2 ($z<0$, substrate). To solve the eigenmode propagating along a direction having an angle $\varphi$ with respect to the $y$ axis, we define a new $x^{'}y'z$ coordinates to be the original $xyz$ coordinates rotated by an angle $\varphi$ in the $xy$ plane; this way, the eigenmode propagates exactly along the $x'$ direction. In the following, we match the boundary conditions in the $x^{'}y^{'}z$coordinates.

Within the frame of *k*DB system (*39*), the surface conductivity $\sigma'$ in the $x^{'}y'z$ coordinates can be expressed as:

$\sigma^{'}=T\cdot\sigma\cdot T^{-1}=\left( \begin{matrix} \sigma_{xx}sin\varphi^{2}+\sigma_{yy}cos\varphi^{2} & {(\sigma}_{xx}-\sigma_{yy})sin\varphi cos\varphi& 0 \\ {(\sigma}_{xx}-\sigma_{yy})sin\varphi cos\varphi& \sigma_{xx}cos\varphi^{2}+\sigma_{yy}sin\varphi^{2} & 0 \\ 0 & 0 & 0 \end{matrix} \right)$ (1)

where $T=\left( \begin{matrix} sin\varphi& -cos\varphi& 0 \\ cos\varphi& sin\varphi& -sin\varphi\\ 0 & 0 & 1 \end{matrix} \right)$, $T\cdot T^{-1}=I$ and $I$ is the unitary matrix.

For the hybrid TM-TE eigenmode, its total field can be written as the summation of field components of pure TM waves and field components of pure TE waves. Without loss of generality, a coefficient $\alpha$ is assumed for TE field components. Then we have

$\bar{H}_{total}=\bar{H}_{TM}+\alpha\bar{H}_{TE}$ (2)

$$\bar{E}_{total}=\bar{E}_{TM}+{\alpha\bar{E}}_{TE}$$

For TM waves, in the $x'y'z$ coordinates, the fields in each region can be expressed as

$$\bar{H}_{TM,1}=\hat{y}'e^{ik_{x^{'}}x^{'}+k_{z_{1}}z}$$

$$\bar{E}_{TM,1}=\frac{-1}{\omega\varepsilon_{0}\varepsilon_{r1}}\left( k_{x^{'}}\hat{z}+ik_{z_{1}}\hat{x}' \right)e^{ik_{x^{'}}x^{'}+k_{z_{1}}z}$$

$\bar{H}_{TM,2}=\hat{y}'\cdot Ae^{ik_{x^{'}}x^{'}-k_{z_{2}}z}$ (3)

$\bar{E}_{TM,2}=\frac{-1}{\omega\varepsilon_{0}\varepsilon_{r2}}\bar{k}_{2}\times\bar{H}_{2}=\frac{-A}{\omega\varepsilon_{0}\varepsilon_{r,2}}\left( \hat{z}k_{x^{'}}-ik_{z_{2}}\hat{x}' \right)e^{ik_{x^{'}}x^{'}-k_{z_{2}}z}$

For TE waves, in the $x'y'z$ coordinates, the fields in each region can be expressed as

$$\bar{E}_{TE,1}=\hat{y}'{\alpha e}^{ik_{x^{'}}x^{'}+k_{z_{1}}z}$$

$$\bar{H}_{TE,1}=\frac{\alpha}{\omega\mu_{0}}\left( k_{x^{'}}\hat{z}+ik_{z_{1}}\hat{x}' \right)e^{ik_{x^{'}}x^{'}+k_{z_{1}}z}$$

$\bar{E}_{TE,2}=\hat{y}'\cdot{\alpha Be}^{ik_{x^{'}}x^{'}-k_{z_{2}}z}$ (4)

$\bar{H}_{TE,2}=\frac{\alpha B}{\omega\mu_{0}}\left( k_{x^{'}}\hat{z}-ik_{z_{2}}\hat{x}' \right)e^{ik_{x^{'}}x^{'}-k_{z_{2}}z}$

In the above equations, $k_{z_{j}}=\sqrt{\frac{\omega^{2}}{c^{2}}\varepsilon_{rj}-k_{x^{'}}^{2}-k_{y^{'}}^{2}}$ is the vertical wavevector component and $\varepsilon_{rj}$ ($j=1\mathrm{or} 2$) are the relative permittivities of regions 1 and 2, respectively. The boundary conditions at $z=0$ require $\hat{n}\times\left( \bar{E}_{1}-\bar{E}_{2} \right)=0$and $\hat{n}\times\left( \bar{H}_{1}-\bar{H}_{2} \right)=\bar{J}_{s}$, where $\hat{n}=-\hat{z}$. By solving the boundary conditions, we have

$\left[ 1+\frac{k_{z1}\varepsilon_{r2}}{k_{z2}\varepsilon_{r1}}+(\sigma_{xx}{sin}^{2}\varphi+\sigma_{yy}{cos}^{2}\varphi)\frac{k_{z1}}{\omega\varepsilon_{0}\varepsilon_{r1}} \right]=\frac{\left( \sigma_{xx}-\sigma_{yy} \right)^{2}{sin}^{2}\varphi{cos}^{2}\varphi\cdot\frac{ik_{z_{1}}}{\omega\varepsilon_{0}\varepsilon_{r1}}}{\sigma_{xx}{cos}^{2}\varphi+\sigma_{yy}{sin}^{2}\varphi+\frac{(k_{z1}+k_{z2})}{\omega\mu_{0}}}$ (5)

where ${sin}^{2}\varphi=\frac{k_{x}^{2}}{k_{x}^{2}+k_{y}^{2}}, {cos}^{2}\varphi=\frac{k_{y}^{2}}{k_{x}^{2}+k_{y}^{2}}$. For the highly squeezed polaritons studied in this work, we show that equation (5) can be approximately reduced to

$\left[ 1+\frac{k_{z1}\varepsilon_{r2}}{k_{z2}\varepsilon_{r1}}+(\sigma_{xx}{sin}^{2}\varphi+\sigma_{yy}c{os}^{2}\varphi)\frac{k_{z1}}{\omega\varepsilon_{0}\varepsilon_{r1}} \right]=0$ (6)

This is because $\left| \frac{\left( \sigma_{xx}-\sigma_{yy} \right)^{2}{sin}^{2}\varphi{cos}^{2}\varphi\cdot\frac{ik_{z_{1}}}{\omega\varepsilon_{0}\varepsilon_{r1}}}{\sigma_{xx}{cos}^{2}\varphi+\sigma_{yy}{sin}^{2}\varphi+\frac{i\left( k_{z1}+k_{z2} \right)}{\omega\mu_{0}}} \right|\approx\left| \frac{-\left( \sigma_{xx}-\sigma_{yy} \right)^{2}\sin^{2} \phi\cos^{2} \phi}{\frac{2\varepsilon_{0}\varepsilon_{r1}}{\mu_{0}}} \right|\ll1$ for highly squeezed polaritons. Equations (5-6) indicate that the dispersion of hybrid TM-TE polaritons can be approximately governed by the dispersion of pure TM polaritons.

**Section S2: All-angle negative refraction of hyperbolic graphene plasmons**

Following Ref. (*20*), the surface conductivity of graphene monolayer is modelled by the Kubo formula (*49*), i.e.,

$\sigma_{s}=\frac{ie^{2}k_{B}T}{\pi\hbar^{2}\left( \omega+i/\tau\right)}(\frac{\mu_{c}}{k_{B}T}+2ln(e^{-\mu_{c}/k_{B}T}+1))+\frac{ie^{2}(\omega+i/\tau)}{\pi\hbar^{2}}\int_{0}^{\infty} \frac{f_{d}\left( -x \right)-f_{d}(x)}{{(\omega+i/\tau)}^{2}-4{(x/\hbar)}^{2}}dx$ (7)

where $f_{d}\left( x \right)={(e^{(x-\mu_{c})/k_{B}T}+1)}^{-1}$ is the Fermi-Dirac distribution; $k_{B}$ is the Boltzmann’s constant; $\mu_{c}$ is the chemical potential; $T=300$ K is the temperature; $\tau=\mu_{c}\mu/(ev_{F}^{2})$ is the relaxation time; $v_{F}=1\times{10}^{6}$ m/s is the Fermi velocity; $e$ is the elementary charge. In this work, a conservative electron mobility of $\mu=10000$ cm^2^V^-1^s^-1^ (*36, 37*) is adopted.

From the main text, the effective anisotropic surface conductivity of graphene metasurface can be described by $\sigma_{xx,l}=\frac{L\sigma_{s}\sigma_{C}}{W\sigma_{C}+\left( L-W \right)\sigma_{s}}$and $\sigma_{yy,l}=\sigma_{s}\frac{W}{L}$, where $W$ is the width of nanoribbon, $\sigma_{C}=-i(\omega\varepsilon_{0}L/\pi)ln[csc(\pi(L-W)/2L)]$ is an equivalent conductivity associated with the near-field coupling between adjacent nanoribbons. By using the setup in the main text, (i.e., the nanostructured graphene has a chemical potential of 0.1 eV, a pitch of $L=30$ nm, and a width of $W=20$ nm), the real parts of the effective surface conductivity of graphene metasurface are shown in Fig. S1. We note $Re\left( \sigma_{yy,l} \right)\gg Re\left( \sigma_{xx,l} \right)$ and $Re\left( \sigma_{xx,l} \right)\ll G_{0}$. The imaginary parts of the effective surface conductivity of graphene metasurface are shown in Fig. 2A.

**
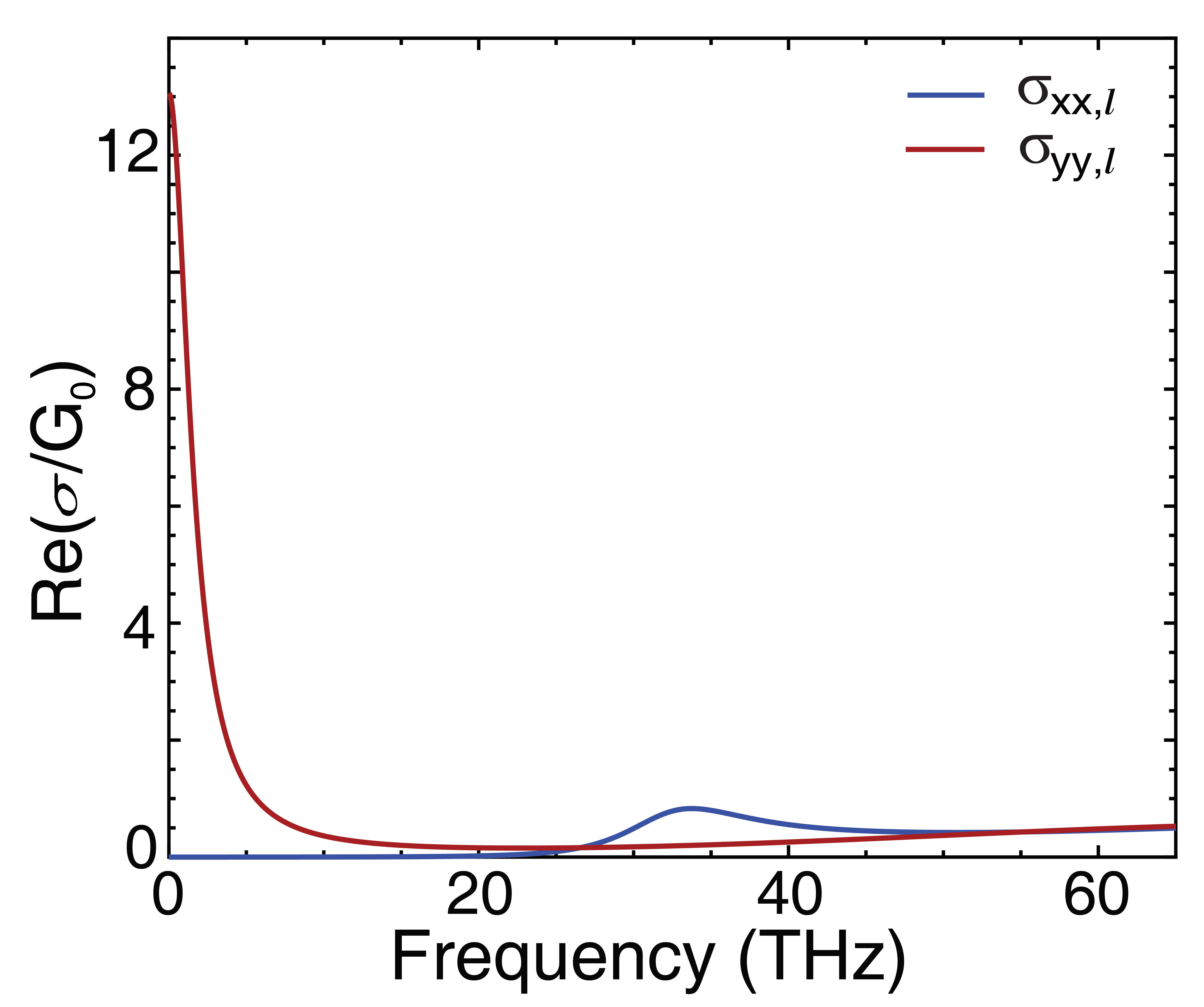
**

**Fig. S1.** **Real part of effective surface conductivity of graphene metasurface.** The setup of graphene metasurface is the same as that in Fig. 2A.

For the clarity of conceptual demonstration, the value of $Re(\sigma_{yy,l})$ is artificially set to be equal to$Re(\sigma_{xx,l})$ in Figs.1C&3. We note that the FWHM (full width at half maximum) of the image for the point source in Fig. 1C is only 0.035 μm, which is less than 1/100 of the working wavelength (i.e., 20 μm) in free space; see Fig. S2. This indicates that the highly squeezed hyperbolic polaritons can enable deep-subwavelength imaging.


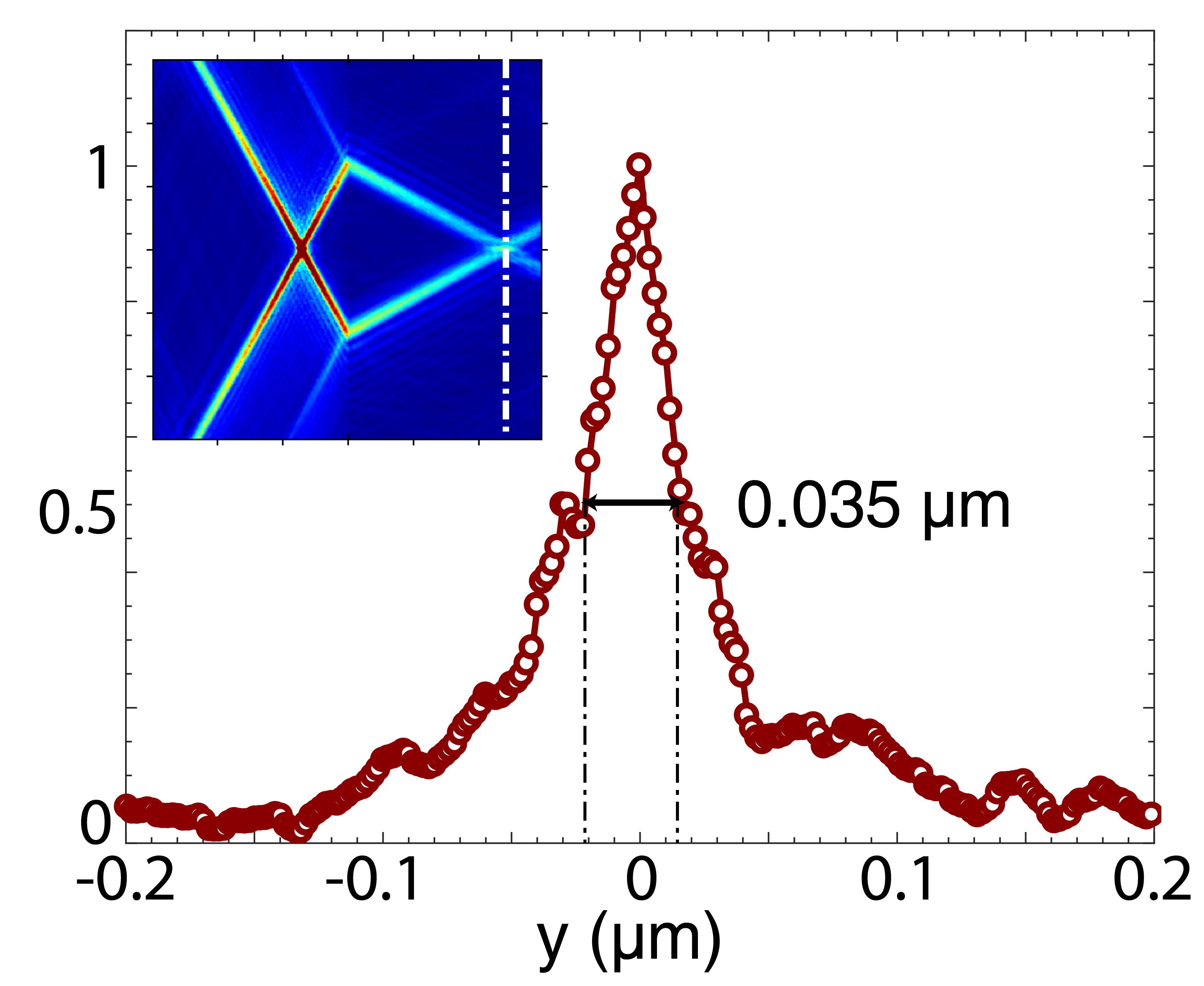


**Fig. S2.** **Full width at half maximum of the image for the point source in Fig. 1C.** The plotted electric field is along a line crossing the center of the image for the point source in Fig. 1C; see the dashed line in the inset. All setup are the same as Fig. 1C and the working wavelength in free space is 20 μm.

To get a vivid understanding of the loss influence, we show the phenomenon of all-angle negative refraction with the consideration of realistic material loss in Fig. S3. The material loss will degrade the propagation length of the hyperbolic graphene plasmons and thus the performance of all-angle negative refraction.

**

Fig. S3 All-angle negative refraction of hyperbolic polaritons when the real material loss is considered.** The working frequency is (**A**) 10 THz, (**B**) 15 THz and (**C**) 20 THz, respectively. The other parameters are the same as that in Fig. 1C.

In addition, the isofrequency contours of hyperbolic graphene plasmons, supported by metasurfaces in the left region in Fig. 1A, are shown in Fig. S4 for different frequencies. We can see from Fig. S4 that the squeezing factor $k_{\rho}/(\omega/c)=\sqrt{k_{x}^{2}+k_{y}^{2}}/(\omega/c$*)* is larger than 100 at the studied frequency range.

**
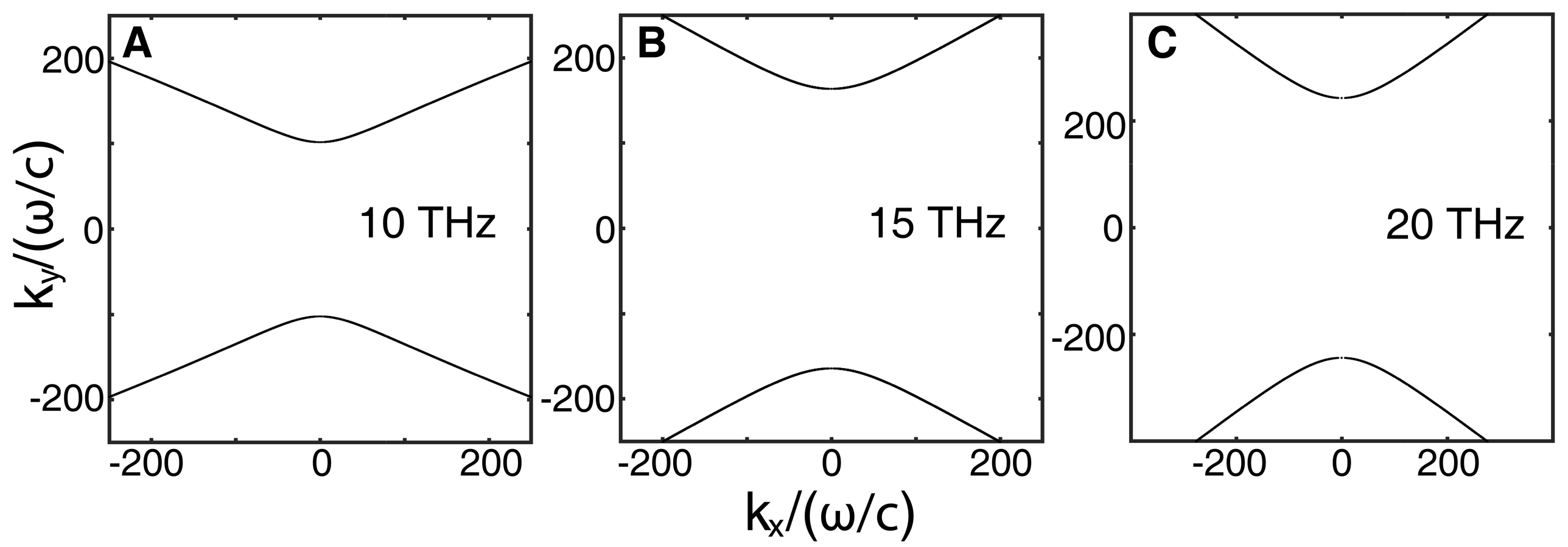
**

**Fig. S4.** **Isofrequency contours of hyperbolic graphene plasmons**. The working frequency is (**A**) 10 THz, (**B**) 15 THz and (**C**) 20 THz, respectively. The hyperbolic graphene plasmons are supported by metasurfaces shown in the left region in Fig. 1A. All parameter setup are the same as Fig. 1B.

Since the negative refraction of graphene plasmons in this work is enabled by the hyperbolic isofrequency contour of graphene plasmons, which exists below 48 THz for the case in Fig. 2A (see the hyperbolic isofrequency contours at 1 THz and 40 THz in Fig. S5 for example), it is reasonable to argue that the negative refraction of hyperbolic polaritons exists below 48 THz for the case in Fig. 2A.


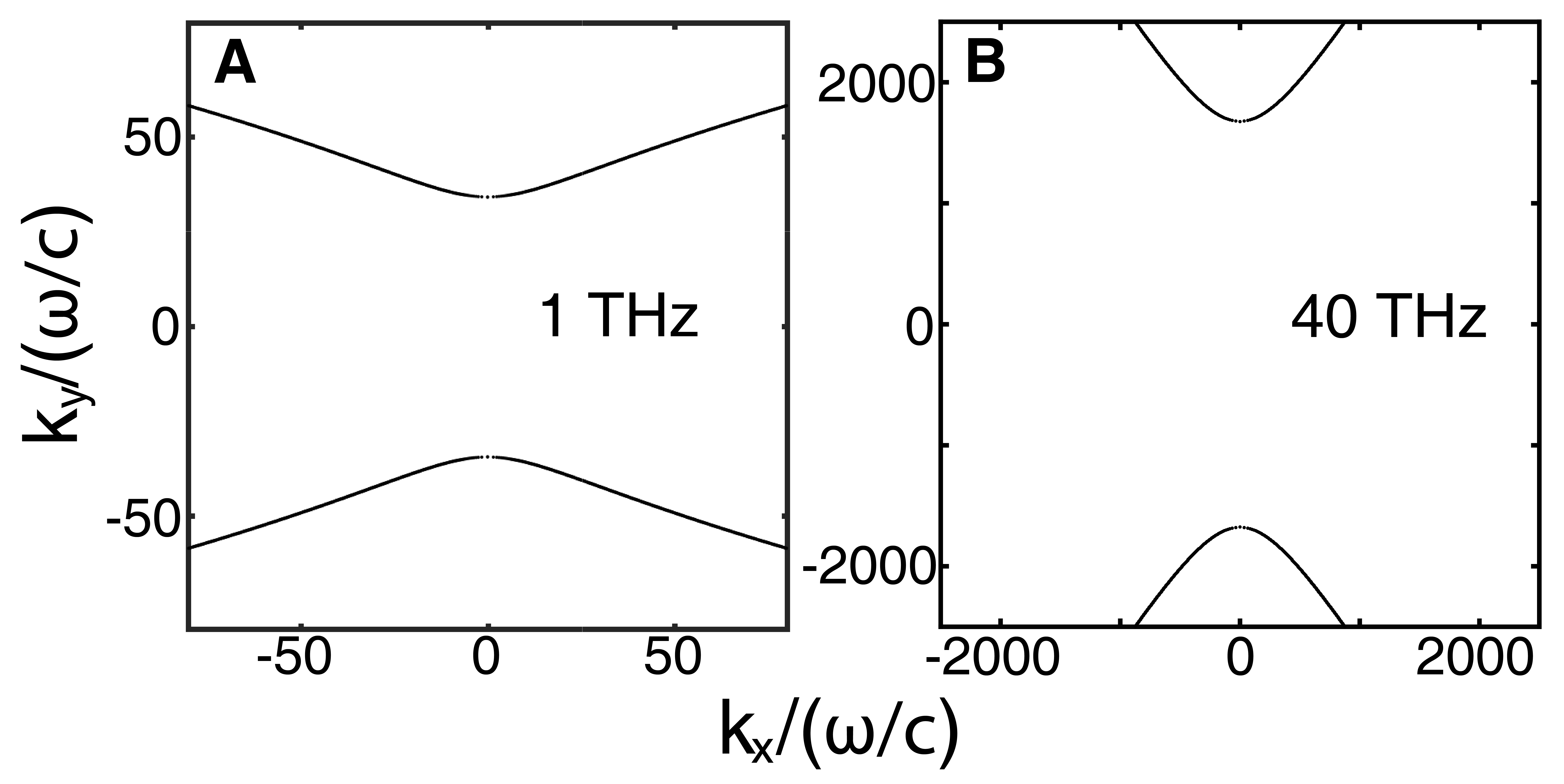


**Fig. S5.** **Isofrequency contours of hyperbolic graphene plasmons at 1 THz and 40 THz.** The working frequencies are (A) 1 THz and (B) 40 THz, respectively. All parameter setup is the same as Fig. 1B in the main text.

Finally, Fig. S6 shows that the change of permittivity of substrate has small influence on the performance of the all-angle negative refraction of hyperbolic graphene plasmons. This gives us the flexibility in choosing the substrate materials. In this work, the dielectric with a relative permittivity of 3.6 (e.g. SiO_2_) is chosen as the substrate for conceptual demonstration (*42*). For clarity of conceptual demonstration, the substrate loss is assumed to be transparent.

**

**

**Fig S6.** **Substrate influence on the all-angle negative refraction of hyperbolic graphene plasmons at 15 THz.** The value of relative permittivity of the substrate is 1 in (**A**), 3.6 in (**B**) and 5.3 in (**C**), respectively. All other parameter setup are the same as Fig. 1C and Fig. 3 in the main text.

In addition, the nanostructures of patterned 2D materials with a pitch of 30 nm proposed in Fig. 1C shall be feasible (although challenging) in experiments. Recently, the nanostructures of patterned 2D materials with a pitch of 35 nm has been experimentally reported in Ref. (*23*), i.e., Small 14, 1800072 (2018), via high-resolution ion beams. In addition, the negative refraction of hyperbolic polaritons can also exist in nanostructures of patterned 2D materials with a pitch much larger than 30 nm (e.g., a pitch of 100 nm in Fig. S7). Such a large pitch ($\geq100$ nm) for these patterned 2D materials shall make their fabrication not a problem anymore.


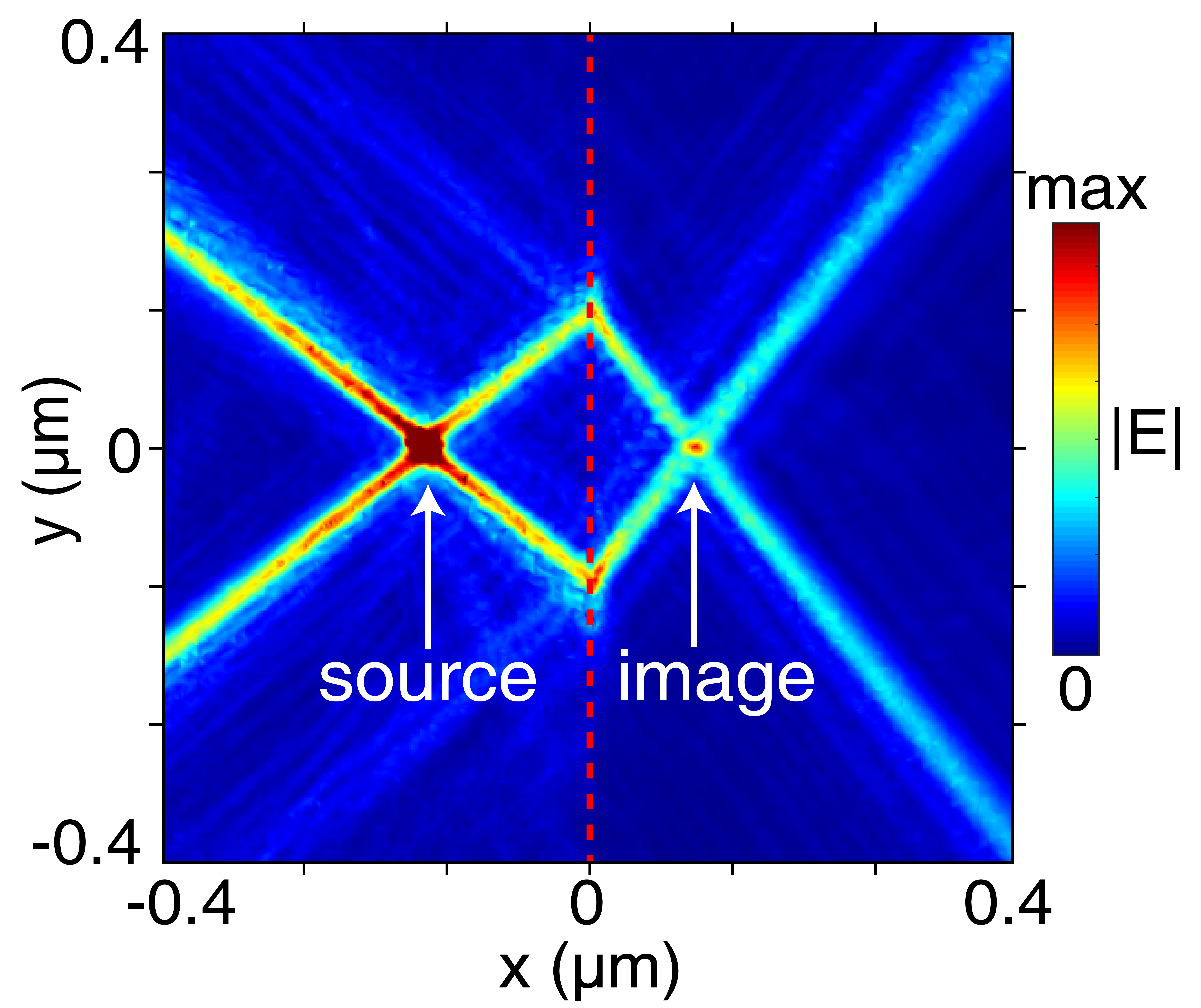


**Fig. S7.** **All-angle negative refraction of hyperbolic polaritons in nanostructures of patterned 2D materials with a pitch of** $\boldsymbol{L}\mathbf{=100}$**nm.** The width of graphene ribbon is $W=70$ nm. The working frequency is 15 THz. The other parameters are the same as Fig. 1C.

**Section S3: Loss influence on the bandwidth having** $\boldsymbol{Im}\left( \boldsymbol{\sigma}_{\boldsymbol{xx}} \right)\boldsymbol{\cdot Im}\left( \boldsymbol{\sigma}_{\boldsymbol{yy}} \right)\boldsymbol{<0}$

As illustrated in Fig. 2B, the material loss can increase the bandwidth having $Im\left( \sigma_{xx,l} \right) \cdot Im\left( \sigma_{yy,l} \right)<0$ when $\mu_{c}$ is smaller than 0.18 eV. Figure S8 shows that this is mainly due to the material loss has a strong influence on the sign of value of $Im\left( \sigma_{xx,l} \right)$. When $\mu_{c}$ is large, such as $\mu_{c}=0.2$eV in Fig. S8A, the bandwidth having $Im\left( \sigma_{xx,l} \right) \cdot Im\left( \sigma_{yy,l} \right)<0$ is merely determined by the frequency where the sign of value of $Im\left( \sigma_{xx,l} \right)$ changes from negative to positive. When $\mu_{c}$ decreases to a value near 0.18 eV, such as $\mu_{c}=0.13$eV in Fig. S8B, there will be two separate frequency ranges having $Im\left( \sigma_{xx} \right)\cdot Im\left( \sigma_{yy} \right)<0$. This way, the bandwidth having $Im\left( \sigma_{xx,l} \right) \cdot Im\left( \sigma_{yy,l} \right)<0$ is determined simultaneously by the frequency where the sign of value of $Im\left( \sigma_{xx,l} \right)$ changes from negative to positive and the frequency where the sign of value of $Im\left( \sigma_{xx,l} \right)$ changes from positive to negative. It is the appearance of the additional frequency range having $Im\left( \sigma_{xx,l} \right) \cdot Im\left( \sigma_{yy,l} \right)<0$ that increases the total bandwidth having $Im\left( \sigma_{xx,l} \right) \cdot Im\left( \sigma_{yy,l} \right)<0.$ When $\mu_{c}$ further decreases, such as $\mu_{c}=0.10$eV in Fig. S8C, the value of $Im\left( \sigma_{xx,l} \right)$ is always negative in the interested frequency range; this way, the bandwidth having $Im\left( \sigma_{xx,l} \right) \cdot Im\left( \sigma_{yy,l} \right)<0$ becomes to be determined by the frequency where the sign of value of $Im\left( \sigma_{yy,l} \right)$ changes from positive to negative. As a summary, we plot the values of $\sigma_{xx,l}$ and $\sigma_{yy,l}$ as a function of $\mu_{c}$ and frequency in Fig. S8D-H. The results in Fig. S8D-H is in accordance with the analysis in Fig. S8A-C.

**

**

**Fig. S8.** **Loss influence on the bandwidth having** $\boldsymbol{Im}\left( \boldsymbol{\sigma}_{\boldsymbol{xx,l}} \right) \boldsymbol{\cdot Im}\left( \boldsymbol{\sigma}_{\boldsymbol{yy,l}} \right)\boldsymbol{<0}$**.** (**A-C**) Effective surface conductivity of graphene metasurface at different chemical potentials. The region having $Im\left( \sigma_{xx,l} \right) \cdot Im\left( \sigma_{yy,l} \right)<0$ is highlighted by light yellow. (**D-H**) Effective surface conductivity of graphene metasurface as a function of the chemical potential $\mu_{c}$ and the frequency. All other parameter setup is the same as Fig. 2A. The red lines in (D, E) indicate that the value of $Im\left( \sigma_{xx,l} \right)$ or $Im\left( \sigma_{yy,l} \right)$ is zero. The line with square symbol is the same as the line of bandwidth having $Im\left( \sigma_{xx,l} \right) \cdot Im\left( \sigma_{yy,l} \right)<0$ as a function of $\mu_{c}$ in Fig. 2B. (D, E) show that the bandwidth having $Im\left( \sigma_{xx,l} \right) \cdot Im\left( \sigma_{yy,l} \right)<0$ is determined by the frequency having $Im\left( \sigma_{xx,l} \right)=0$ when $\mu_{c}>0.2$ eV, and becomes determined by the frequency having $Im\left( \sigma_{yy,l} \right)=0$ when $\mu_{c}<0.12$ eV.
